# Supplementary material for: Forward genetics reveals microsporidian drug targets and recombination-based resistance
Source: NPJ Antimicrob Resist. 2026 Jul 3;4:77. doi: 10.1038/s44259-026-00245-0 (PMC13745600; doi:10.1038/s44259-026-00245-0)
Supplement: Supplementary file 1 [file 44259_2026_245_MOESM1_ESM.pdf]

Supplementary materials

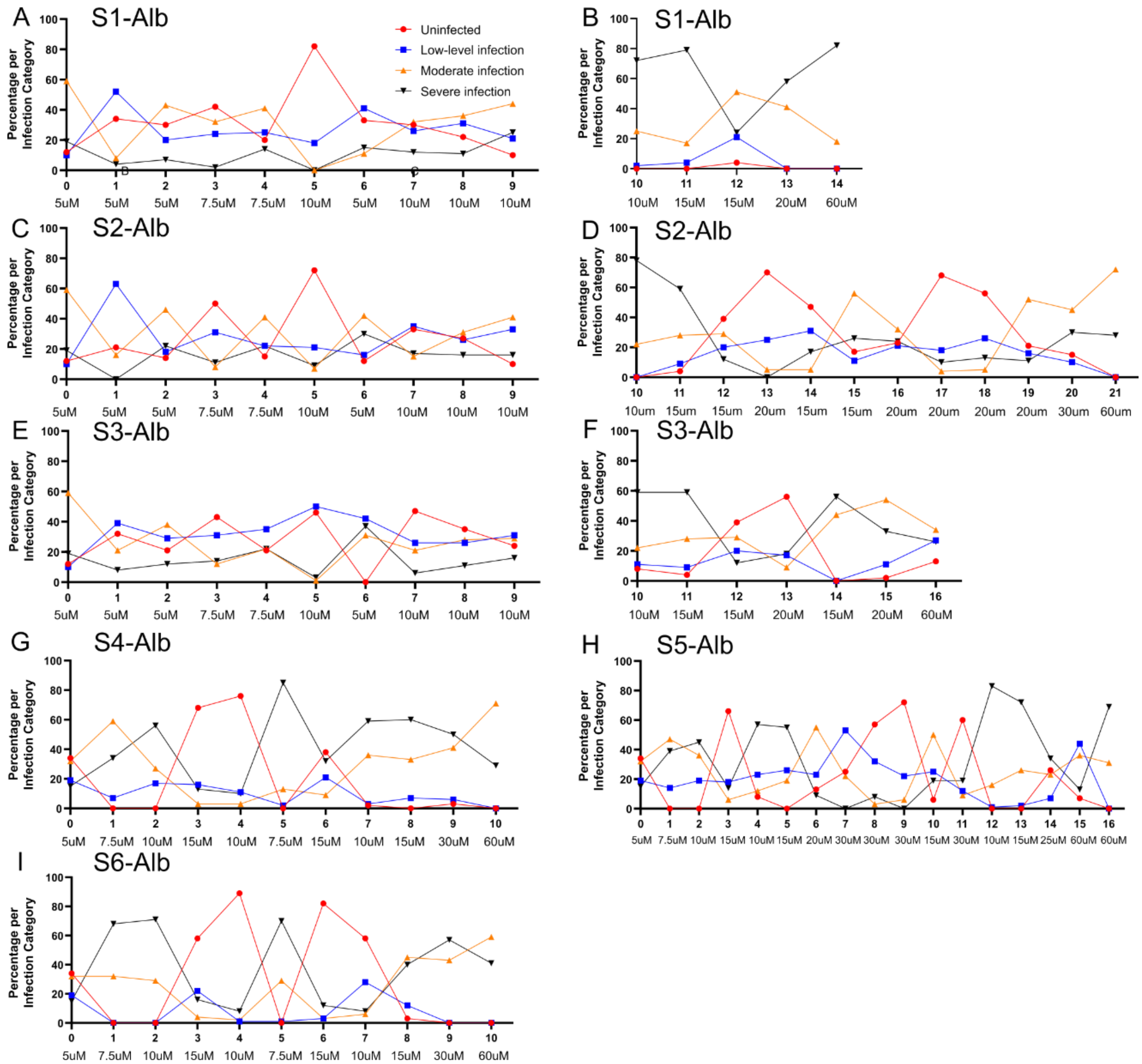

Figure S1. Relationship between drug concentration and infection severity in albendazole-resistant spore selections over multiple generations. A-I. For the initial (A, C, E; incubated at 21°C for 4 days) and optimized (B, D, F, G, H, I; incubated at 25°C for 6 days) protocols, a co-culture was established by adding 1 mL of infected nematode culture to 49 mL of 2× S-complete containing 10,000 L1 larvae. Infection status was assessed using DY96 and DAPI staining. The albendazole concentration was decreased if >80 % of adults were uninfected, increased if >50 % showed moderate to severe infection, and otherwise maintained. Cultures exhibiting >50 % moderate to severe infection at 60 μM albendazole were considered to harbor resistant spores and were subsequently validated by a 24-well continuous infection assay. Generation number is shown in bold under the x-axis along with the albendazole concentration.

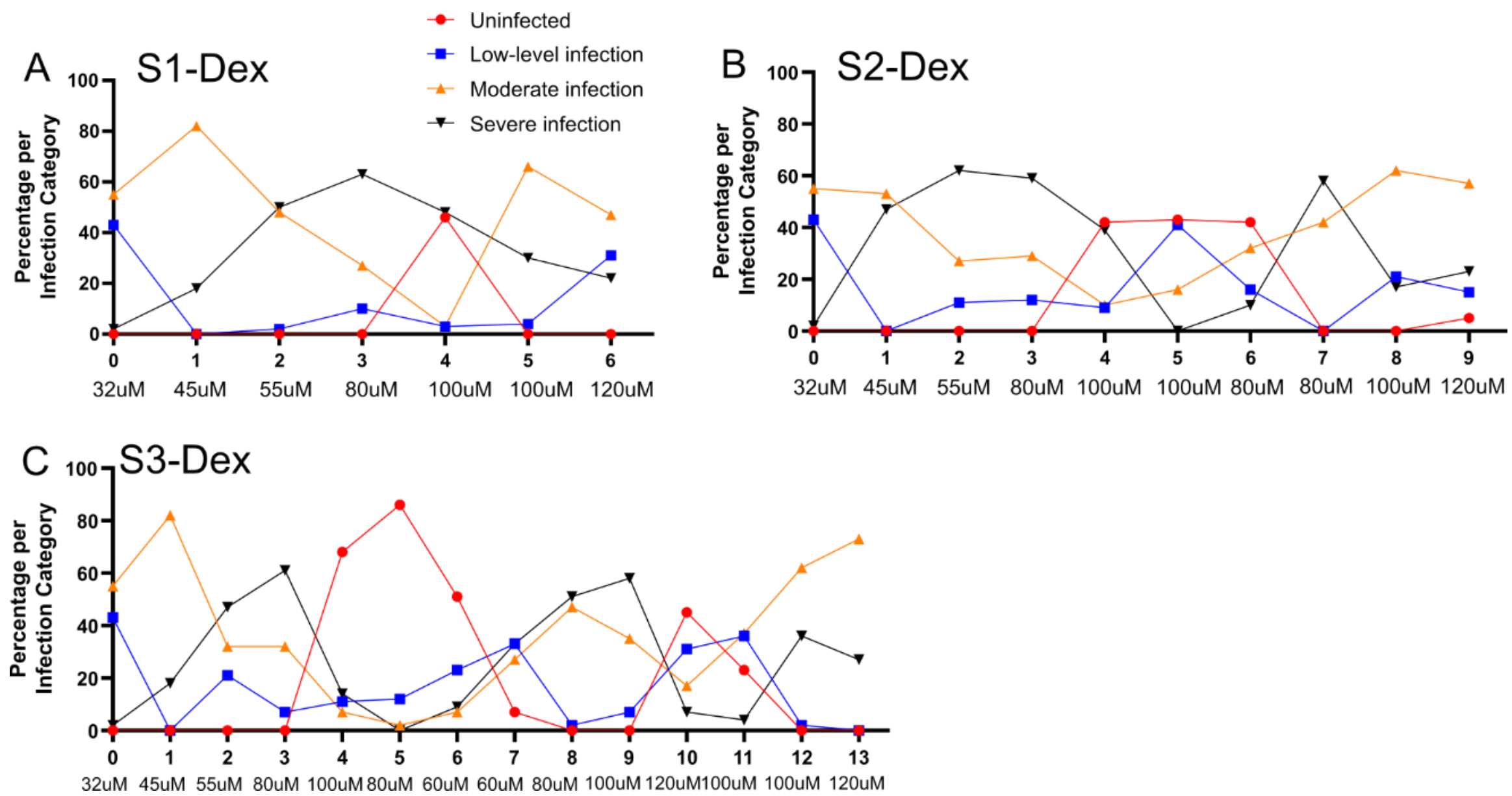

Figure S2. Relationship between drug concentration and infection severity in dexrazoxane-resistant spore selections over multiple generations. A-C. Application of the optimized resistant spore screening protocol. A co-culture was established by adding 1 mL of infected nematode culture to 49 mL of 2× S-complete containing 10,000 L1 worms, followed by incubation at 25 °C for 6 days. Infection status was assessed using DY96 and DAPI staining. The dexrazoxane concentration was decreased if >80% of adult worms were uninfected, increased if >50% exhibited moderate-to-severe infection, and maintained otherwise. Cultures showing >50% moderate-to-severe infection at 120 μM dexrazoxane were considered to harbor resistant spores and proceeded to validation by 24-well continuous infection assay. Generation number is shown in bold under the x-axis along with the dexrazoxane concentration.

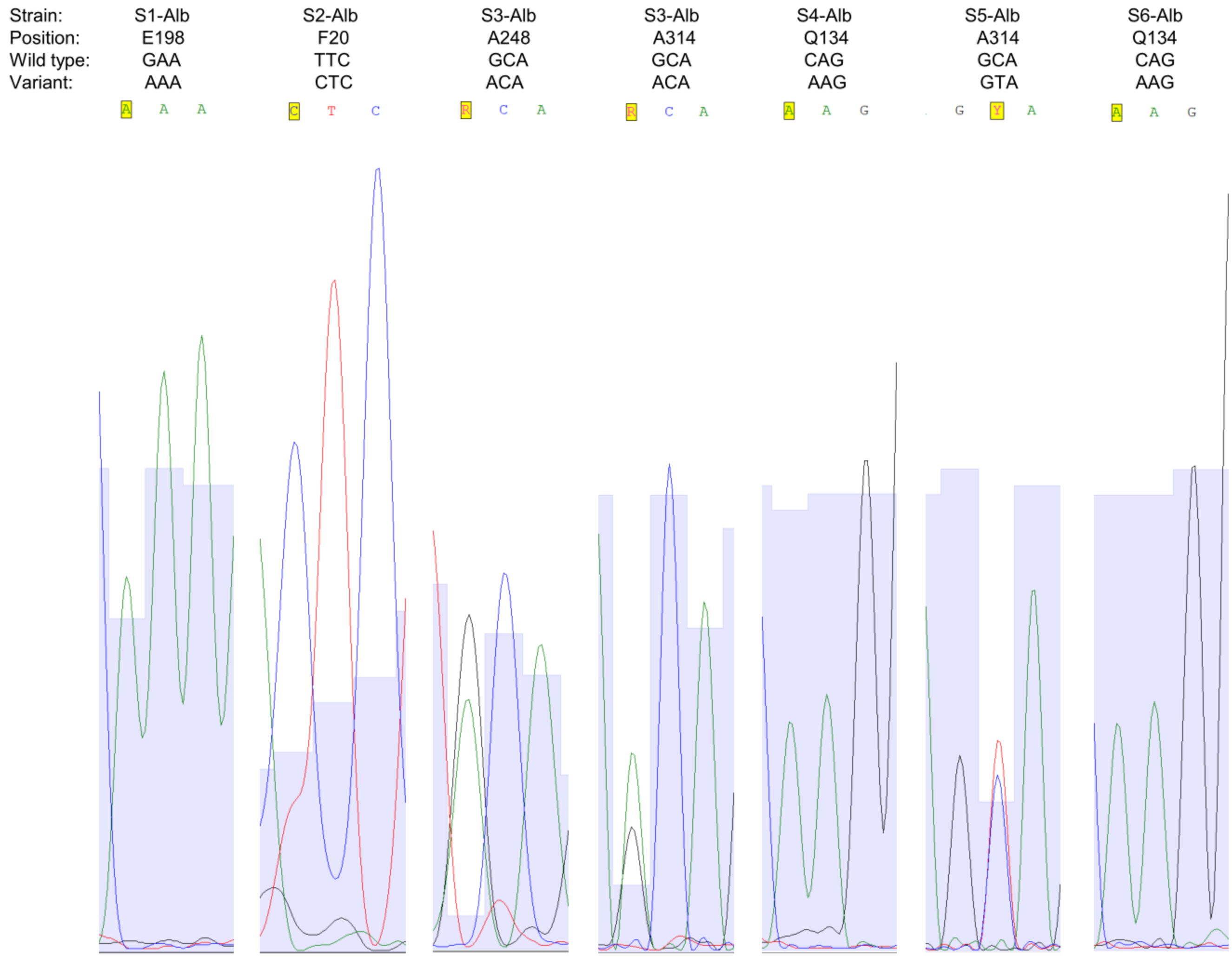

Figure S3. Sanger-sequencing validation of beta-tubulin mutations in albendazole-resistant *N. parisii* strains. The beta-tubulin gene from the albendazole-resistant strains was amplified using PCR and Sanger sequenced. The sequencing chromatograms for the codon containing the variants detected by whole genome sequencing are shown for each strain. The bases are colored with adenine in green, cytosine in blue, thymine in red, and guanine in black. The base changed in the resistant strains is highlighted in yellow. The position, the ERTm1 wild-type codon, and the variant observed in the resistant strain are shown above the chromatograms. APE was used to visualize chromatograms [65].

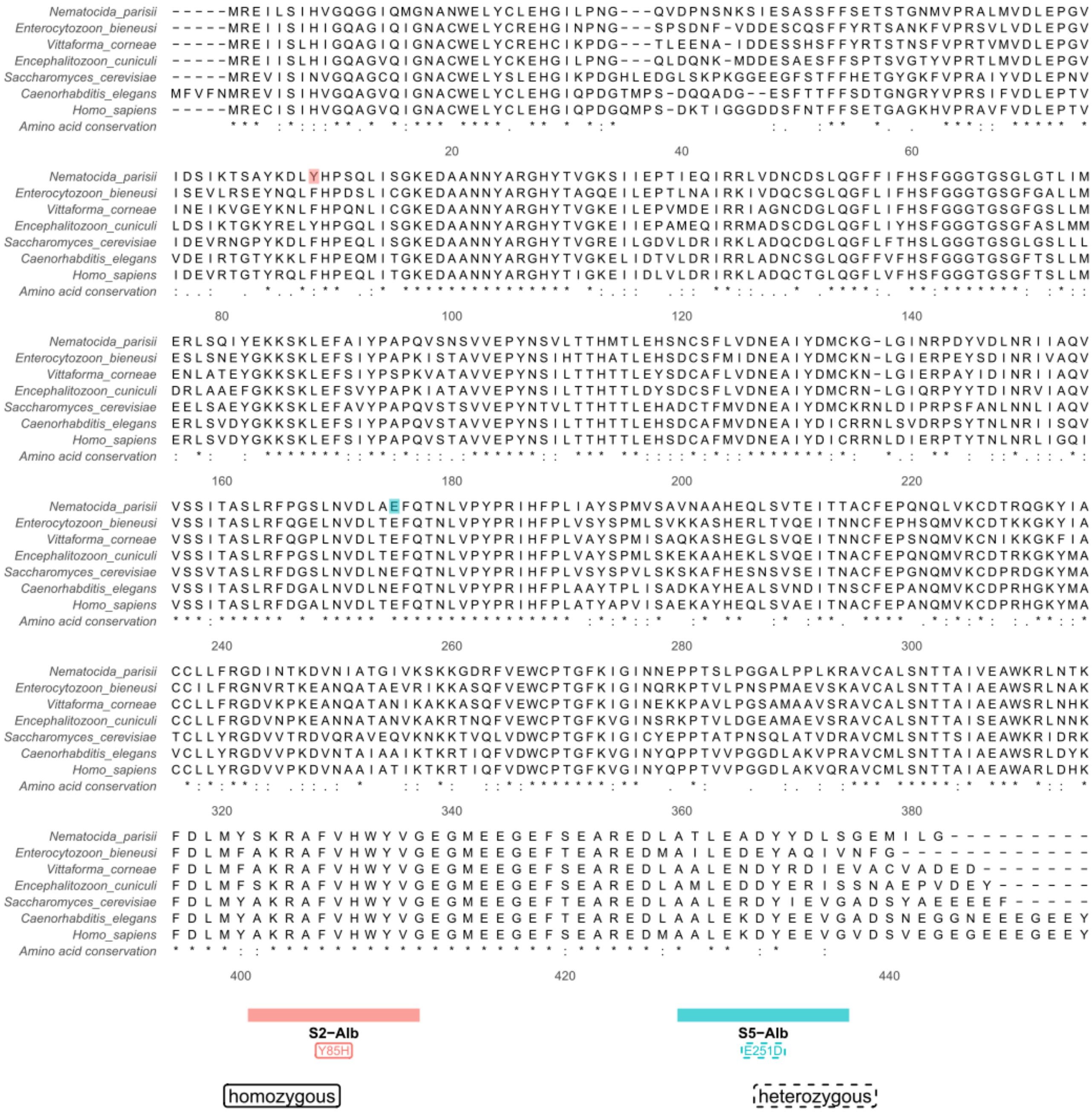

Figure S4. Two albendazole-resistant *N. parisii* strains contain either homozygous or heterozygous variants in alpha-tubulin. Multiple sequence alignment of alpha-tubulin sequences from microsporidia *(N. parisii*, *E. bieneusi*, *V. corneae*, and *E. cuniculi)*, *S. cerevisiae*, *C. elegans*, and humans. Homozygous and heterozygous variants that were detected in two of the albendazole-resistant strains are annotated according to the legend at the bottom. Positions are numbered according to *S. cerevisiae* TUB1 and amino acid conservation is shown at the bottom of the alignment.

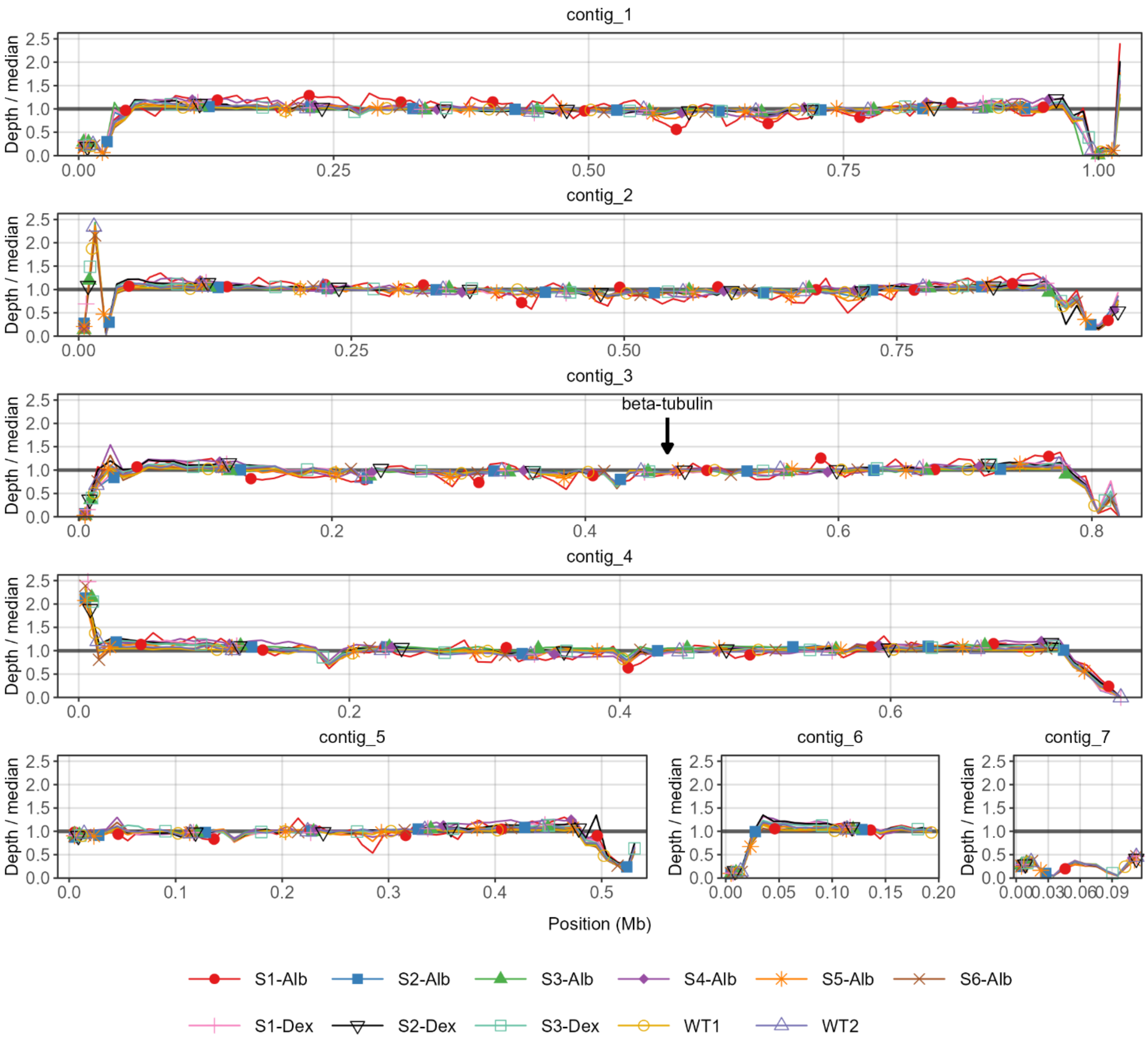

Figure S5. Read depth in drug-resistant *N. parisii* strains does not support aneuploidy. The read depth across the *N. parisii* genome assembly was determined for the nine drug-resistant *N. parisii* strains and two wild-type ERTm1 (WT1 and WT2) samples. The read depth at 10 kb windows divided by the median read depth for each sample at each contig is shown. The location of the beta-tubulin locus on contig 3 is indicated with an arrow. The identity of the strains is indicated by the legend at the bottom.

Table S1. ***N. parisii*** **homozygous and heterozygous variants with predicted protein-coding impacts that occur in albendazole- or dexrazoxane-resistant strains.**

Table S2. ***N. parisii*** **homozygous and heterozygous variants in intergenic regions that occur in albendazole- or dexrazoxane-resistant strains.**

Data S1. All infection data from the study.
